# Trait xenophobia is more strongly related to maladaptive beliefs and pandemic health behaviors than affective symptoms

**DOI:** 10.3389/fpsyg.2025.1614848

**Published:** 2025-11-10

**Authors:** Michael J. Minzenberg, Jong H. Yoon

**Affiliations:** 1 Department of Psychiatry, School of Medicine, University of California, San Diego, San Diego, CA, United States; 2 Department of Psychiatry, Stanford University School of Medicine, Palo Alto, CA, United States

**Keywords:** xenophobia, conspiracy theories, paranoia, anxiety, depression, pandemic, health behavior

## Abstract

**Background:**

Xenophobia is a prevalent phenomenon with significant personal and societal consequences. As expressed by individuals, it can be influenced by psychological or psychiatric factors. Negative affect is an important aspect of xenophobia, and both negative affect and xenophobia have increased in prevalence during the COVID-19 pandemic, suggesting a potential association. We tested whether trait-like xenophobic beliefs relate to common affective symptoms, compared to other maladaptive beliefs such as conspiratorial beliefs, and, in turn, how these symptoms and beliefs relate to pandemic-related health behaviors.

**Methods:**

A total of 520 American adults completed validated online self-reported questionnaires addressing xenophobia, conspiracy beliefs, symptoms of paranoia, anxiety and depression, fear of COVID-19, and inclination to engage in pandemic-related behaviors (precautions, testing, and vaccination).

**Results:**

Statistically significant positive bivariate correlations of moderate strength were observed between xenophobia and conspiracy beliefs, both general (conspiracy-mindedness) and specific (about vaccines). Significant but weaker positive correlations were observed between xenophobia and paranoia, anxiety, and depression. An exploratory factor analysis of symptoms and beliefs revealed a two-factor solution accounting for 66% of the total variance. Xenophobia had a strong loading on the Maladaptive Belief factor, with factor scores significantly negatively associated with the three pandemic health behaviors, whereas Psychopathology factor scores showed weaker, positive associations with pandemic health behaviors. Regression models of pandemic health behaviors similarly showed both factor scores as significant independent predictors of each health behavior, with Maladaptive Belief contributing a relatively much larger share of variance.

**Conclusion:**

Xenophobia is more strongly associated with other maladaptive beliefs than with common psychiatric symptoms. Maladaptive beliefs, compared with common psychiatric symptoms, is also more closely associated with a disinclination to engage in adaptive pandemic-related health behaviors. These findings may have implications for mitigating the personal and public health effects of a global pandemic.

## Introduction

Xenophobia is a phenomenon of social (outgroup) antagonism that has been defined in various ways, such as “an attitudinal orientation of hostility against non-natives in a given population,” from the International Migration, Racism, Discrimination, and Xenophobia Conference held by multiple international human rights groups under the auspices of the United Nations (Boehnke, 2001). Other definitions have similarly emphasized negative affective states as an important element, most commonly the disgust, fear, and hatred toward outgroup members. The impact of xenophobia is massive, serving a crucial foundation for much of the most horrific phenomena in human history, such as warfare, terrorism, slavery, and genocide (Bogerts and Steinmetz, 2021). In healthcare settings, xenophobia has been associated with poor access to and outcomes of care. For instance, during the course of infectious outbreaks and pandemics, the stigmatizing effects of xenophobia have been associated with the emergence of critical inequalities in various dimensions (e.g., health, economic, social, and political), heightened state control of foreigners in some countries (such as mandatory health screening specific to foreigners), reduced healthcare access (Silva et al., 2022), and worse medical outcomes, including mental health (Suleman et al., 2022).

Many observers have noted a rise in xenophobic attitudes and policies during the COVID-19 pandemic (Taylor, 2022). This rise has been evident globally in community settings (Reny and Barretto, 2022, and Taylor, 2022), healthcare settings (Huang and Liu, 2020), and even in policy prescriptions for the handling and sales of food items (Chuvileva et al., 2020).

The emergence or exacerbation of xenophobia may be a general aspect of pandemics (Kam, 2019; Silva et al., 2022; Taylor, 2022). An influential model that suggests how individuals respond to the threat of contagion with heightened outgroup antagonism is characterized as the Behavioral Immune System (BIS; Schaller et al., 2014). The BIS is comprised of distributed neural circuits (centered on limbic interactions with cortical regions) that have evolved to guide complex behaviors that reduce exposure to potentially dangerous pathogens, which are often those for which the individual has no prior exposure (such as those brought by European colonists to the Americas; Crosby, 1976). This system is typically activated by perceptual cues that form outward expressions of infectious illnesses in others. Critically, the perception of these cues (visual and olfactory) exerts a negative bias and often induces erroneous inferences, the latter of which is a prevalent feature of xenophobic beliefs (Rydgren, 2004). A central role in the operation of the BIS is the generation of negative affect, such as disgust, fear, and hatred, typically directed at the source of perceptual cues. As Kavaliers et al. (2022) note, while social interaction generally facilitates the transmission of information, it may also facilitate the spread of pathogens. This risk–benefit trade-off forms a classic approach-avoidance conflict within an individual. Schaller et al. (2014) emphasize the general principle of *context-contingent flexibility* in BIS mechanisms, where an underlying general human tendency toward xenophobia is manifested especially strongly under conditions in which people perceive a heightened vulnerability to infection. Thus, various links between xenophobia and BIS sensitivity (typically measured as perceived vulnerability to infection), fear of contagion, and disgust have been identified, both as stable trait-like characteristics and beliefs induced by experimental manipulations of these variables (via narratives and perceptual cues) (Faulkner et al., 2004; Landry et al., 2022).

Given the central role of negative affect in both BIS function and xenophobia, the degree to which xenophobia may be linked with affective symptoms remains unclear. Importantly, symptoms of negative affect, such as anxiety, paranoia, and depression, are highly prevalent globally, and these conditions have both diverse origins and varied expressions and impacts on the function of individuals, including social and interpersonal function. In addition, there is evidence that these symptoms have become more prevalent globally since the onset of the COVID-19 pandemic (Suthaharan et al., 2021; Penninx et al., 2022). The American Psychiatric Association (2020) published a laudable position statement on xenophobia, which is notable for addressing the social and health effects on the targets of xenophobia. However, it remains unclear what psychological factors relate to the intensity or persistence of xenophobia among individuals. In light of the relationship between xenophobia and negative affect and the context-dependent association between both xenophobia and negative affect syndromes and the emergence of pandemics and attendant health behavior responses, we tested the link between trait xenophobia and symptoms of common clinical syndromes (anxiety, paranoia, and depression) and health behaviors during the COVID-19 pandemic. We conducted this study using a non-clinical sample to address these relationships in a symptom-based (rather than clinical disorder-based) manner. We tested these potential associations between xenophobia and those of other maladaptive beliefs commonly found in non-clinical populations, specifically conspiratorial beliefs, which are similarly maladaptive for health behaviors, social relationships, and political expression (Douglas et al., 2019). These beliefs are heightened during pandemics (Bogerts and Steinmetz, 2021) and linked to xenophobic attitudes and intergroup conflict (Hebel-Sela et al., 2022; van Prooijen and Song, 2021).

## Materials and methods

We conducted an anonymous, panel-based online survey using the Qualtrics survey service, with sampling conducted between 12 May 2022 and 27 May 2022. This study was determined to be exempt from IRB review (CIRBI, Advarra, Inc.). All respondents were adults located in the United States. Qualtrics maintains a network comprising hundreds of respondent suppliers using diverse recruitment methods (Miller et al., 2020). Respondents were recruited via a double opt-in procedure, and they were typically compensated in cash, gift cards, or reward points. Basic demographic data were obtained using US Census question phrasing, and demographic targeting was achieved by combining quota sampling and screening questions. Educational attainment is obtained using categories “high school,” “some college or vocational training,” “college graduate,” and “graduate school or professional degree.” Qualtrics uses validation checks, e.g., excluding incomplete surveys and excessively “speeded” responses (defined as those with a total completion time < 1/3 of the median time for the full sample), and ensures that each unique IP address can access the survey only once. This survey platform mitigates the problem of “professionalization” of subjects and, in general, meets the “fit for purpose” standard promulgated by the American Association for Public Opinion Research (Baker et al., 2013).

All respondents completed the following self-report instruments (each in English):

Xenophobia Scale (van der Veer et al., 2011). This is a 5-item Likert scale that addresses common expressions of xenophobia. It has been validated in independent samples from several Western nations. Cronbach’s alpha for the current sample was 0.925.

The Conspiracy Mentality Questionnaire (CMQ; Bruder et al., 2013) is a scale measuring trait-like CT-proneness, with five items evaluating how likely respondents believe certain general propositions to be true (not pertaining to specific CTs), each on an 11-point Likert scale ranging from 0% “Certainly not” to 100% “Certainly.” It has excellent convergent, discriminant, predictive, and cross-cultural validity and internal and test–retest reliability (Bruder et al., 2013). Cronbach’s alpha for the current sample was 0.885.

The Vaccine Conspiracy Beliefs Scale (VCBS; Shapiro et al., 2016) is a 7-item Likert-scale that evaluates commonly held conspiracy beliefs about vaccines. Cronbach’s alpha for the current sample was 0.962.

The Patient Health Questionnaire-2 item (PHQ-2; Kroenke et al., 2010) is a 2-item self-report scale evaluating common symptoms of depression, which has wide use in varied healthcare settings and disciplines. Cronbach’s alpha for the current sample was 0.820.

The Generalized Anxiety Disorder-2 item (GAD-2; Sapra et al., 2020; Plummer et al., 2016) is a 2-item self-report scale evaluating common symptoms of anxiety, which has wide use in varied healthcare settings and disciplines. Cronbach’s alpha for the current sample was 0.900.

The Green Paranoid Thoughts Scale—Revised Ideas of Persecution Subscale (R-GPTS; Freeman et al., 2021), a 10-item subscale of the R-GPTS, is a Likert-scale self-report that addresses persecutory ideas common to both clinical and non-clinical populations. Cronbach’s alpha for the current sample was 0.958.

The Fear of COVID-19 Scale (FCV-19S; Ahorsu et al., 2020; Perz et al., 2020) is a 7-item.

Likert scale self-report aimed at detecting fear of COVID in the general population. Cronbach’s alpha for the current sample was 0.926.

In addition, we administered three items (Minzenberg and Yoon, 2022) addressing respondents’ intention in the current COVID-19 pandemic to (A) take precautions, (B) test for COVID-19 infection, and (C) vaccinate against the coronavirus that causes COVID-19 (SARS-CoV-2). The three pandemic items were each scored on an 11-point Likert scale, ranging from “certainly not” to “certainly” (identical to the CMQ Likert response format). We evaluated the intention to vaccinate rather than actual vaccination to minimize variability due to factors outside the respondent’s control, such as access (Azjen, 1991).

Inferential testing was conducted using total scale scores in the following statistical analyses, including Pearson product–moment coefficients among all psychology (i.e., non-demographic) measures and exploratory factor analysis using principal components analysis with the same set of measures. Stepwise linear regression models were also established, entering scores from Factor 2 (derived from PCA) in the first step and Factor 1 in the second step, in order to evaluate the relative proportion of variance in pandemic-related health behaviors contributed by the two factors.

## Results

A total of 520 anonymous adults from the United States completed the questionnaire. The distribution of demographic and clinical variables was as follows:

Sex: 51.3% were female individuals.

Age: 22.1% were 18–24 years old, 34.0% were 35–54 years old, 43.8% were 55 years or older.

Race (using categories from the United States National Institute of Health): 75.0% were white, 13.1% were Black, 6.0% were Asian, 0.8% were native/Indigenous, and 5.2% were mixed race.

Education: 41.9% of participants had a college degree.

Household income: 35.0% earn less than US$50,000 per year, 35.0% earn between US$50,000 and 100,000 per year, and 30.0% earn more than US$100,000 per year.

Descriptive statistics for the non-demographic variables are presented in Table 1. Xenophobia scores showed statistically significant bivariate Pearson correlations with several other measures (the Bonferroni-corrected alpha for each test was *p* = 0.0024). These included correlations of moderate strength with general conspiracy-mindedness (*r* = 0.298, *p* < 0.0005) and especially with specific vaccine conspiracy beliefs (*r* = 0.476, *p* < 0.0005) and relatively lower correlations with paranoia (*r* = 0.219, *p* < 0.0005) and fear of COVID (*r* = 0.154, *p* = 0.001) (Table 2). Correlations between xenophobia and symptoms were generally lower and significant for paranoia and fear of COVID infection, whereas the correlations between anxiety and depression did not survive Bonferroni correction for multiple comparisons.

**Table 1:** Descriptive statistics for variables (total scale scores; all *N* = 520).

	Minimum	Maximum	Mean	SD
Xenophobia	5	35	16.5	8.6
Conspiracy-mindedness	5	55	32.2	11.6
Vaccine conspiracy	7	49	21.7	11.6
PHQ-2	2	8	3.1	1.6
GAD-2	2	8	3.0	1.6
Paranoia	10	50	13.4	7.1
Fear of COVID	7	35	15.1	6.8

**Table 2:** Bivariate correlations (Pearson’s *r*) between xenophobia, conspiratorial belief, and symptom measures (all tabled *p* values < 0.001 uncorrected, except for xenophobia with PHQ-2 [*p* = 0.018] and GAD-2 [*p* = 0.036]).

	Xenophobia	Conspiracy-mindedness	Vaccine conspiracy	PHQ-2	GAD-2	Paranoia	Fear of COVID
Xenophobia		0.30	0.48	0.10	0.09	0.22	0.15
Conspiracy-mindedness	0.30		0.53	0.20	0.23	0.20	0.18
Vaccine conspiracy	0.48	0.53		0.19	0.18	0.28	0.12
PHQ-2	0.10	0.20	0.19		0.79	0.58	0.51
GAD-2	0.09	0.23	0.18	0.79		0.61	0.50
Paranoia	0.22	0.20	0.28	0.58	0.61		0.48
Fear of COVID	0.15	0.18	0.12	0.51	0.50	0.48	

The exploratory factor analysis (principal component analysis) of this dataset yielded the following results: this set was suitable for PCA, with a Kaiser-Meyer-Olkin index of 0.753 and Bartlett’s Test of Sphericity, which yielded a significance level of *p* < 0.001. The PCA revealed a two-component solution using Oblimin rotation with eigenvalues = 3.070 and 1.563, which together accounted for 66.2% of the variance. Anxiety, depression, paranoia, and fear of COVID-19 loaded on the first factor, identified as a Psychopathology factor (Table 3). In contrast, xenophobia, conspiracy-mindedness, and vaccine conspiracy beliefs loaded on the second factor, identified as a Maladaptive Belief factor. Cross-loadings of each measure on the other factor were generally much lower, with only paranoia exceeding 0.30 (loading on Maladaptive Beliefs). Of note, xenophobia loaded on the Psychopathology factor only at 0.15, which was the lowest loading of any variable on either factor.

**Table 3:** Factor loadings of seven measures from exploratory factor analysis and bivariate correlations of factor scores with pandemic health behaviors (* *p* < 0.05, ** *p* < 0.001).

	Factor 1	Factor 2
Xenophobia	0.15	0.75
Conspiracy-mindedness	0.26	0.75
Vaccine conspiracy	0.23	0.86
PHQ-2	0.89	0.19
GAD-2	0.89	0.20
Paranoia	0.79	0.33
Fear of COVID	0.73	0.18
Correlations: factor scores with health behaviors		
COVID precautions	0.11*	−0.30**
COVID testing	0.15**	−0.28**
COVID vaccination	−0.05	−0.47**

Using factor scores in post-hoc bivariate Pearson correlations with COVID-19 pandemic-related health behaviors, Maladaptive Belief factor scores were significantly negatively correlated with all three pandemic behaviors, including precautions, testing, and vaccination (Table 3). In contrast, Psychopathology factor scores showed more modest correlations with precautions and testing, in the opposite direction, and showed no significant correlation with vaccination.

The regression model of COVID precautions was highly statistically significant (*R*^2^ = 0.124, *F* = 36.7, df 2, 517, *p* < 0.001). Both Factor 1 (Psychopathology) and Factor 2 were significant predictors of precautions (both *p* < 0.001; Table 4). The regression model of COVID Testing was highly statistically significant (*R*^2^ = 0.128, *F* = 37.5, df 2, 517, *p* < 0.001). Both Factor 1 (Psychopathology) and Factor 2 were significant predictors of testing (both *p* < 0.001; Table 4). The regression model of COVID vaccination was highly statistically significant (*R*^2^ = 0.223, *F* = 74.18, df 2, 517, *p* < 0.001). Both Factor 1 (Psychopathology) and Factor 2 were significant predictors of vaccination (Factor 1, *p* = 0.038 and Factor 2, *p* < 0.001; Table 4). In each regression model, Factor 2 (Maladaptive Beliefs) contributed a larger share of variance in pandemic-related health behaviors, especially for vaccination.

**Table 4:** Regression model of factor scores from EFA as predictors of pandemic-related health behaviors.

	*R*^2^ change	*B*	Beta	*t*	*p*
COVID precautions					
Factor 2 (Maladaptive Beliefs)	0.088	−1.096	−0.349	−8.176	< 0.001
Factor 1 (Psychopathology)	0.037	0.623	0.198	4.648	< 0.001
COVID testing					
Factor 2 (Maladaptive Beliefs)	0.076	−1.059	−0.338	−7.940	< 0.001
Factor 1 (Psychopathology)	0.052	0.739	0.236	5.540	< 0.001
COVID vaccination					
Factor 2 (Maladaptive Beliefs)	0.216	−1.720	−0.487	−12.122	< 0.001
Factor 1 (Psychopathology)	0.006	0.295	0.084	2.077	0.038

## Discussion

In this study, we conducted a community-based survey to evaluate self-reported xenophobia for its relative strength of association with common symptoms of anxiety, depression, and paranoia. We contrasted this association with that of xenophobia with a parallel, common phenomenon of maladaptive belief found in non-clinical populations, i.e., conspiratorial beliefs, both general (conspiracy-mindedness) and specific (vaccine conspiracy beliefs). Xenophobia was associated with both forms of conspiracy beliefs, and their shared latent factor was significantly associated with a disinclination to engage in three important health behaviors that have been implemented globally to mitigate the effects of the current global pandemic. In contrast, xenophobia was more weakly associated with negative affect, and the Psychopathology factor was weakly associated with three targeted health behaviors.

This survey addressed the common symptoms of negative affect as a potential link between xenophobia and the experience of the current pandemic. As noted above, these symptoms have become more prominent during the course of the pandemic, which may be a ubiquitous consequence of pandemics. Similarly, conspiratorial beliefs may have also increased during the pandemic, with some xenophobic features, and this provided a test of divergence among xenophobia associations (Affect vs. Maladaptive Beliefs).

While the phenomenon of xenophobia has both ideational and affective components, the present results suggest that in the context of the current pandemic, the expression of the ideational component is more strongly linked to other maladaptive phenomena.

Conspiracy theories, in particular, are generally conceived of as having a fundamental component of outgroup antagonism (comprising those who represent the target of the conspiratorial belief), and this intergroup aspect generally distinguishes this type of belief from mere paranoia (van Prooijen and Song, 2021). Furthermore, Hebel-Sela et al. (2022) emphasized the reciprocal nature of violent intergroup conflict and conspiracy theories, where each exacerbates the other, forming a vicious cycle that can spiral out of control, leading to serious and protracted violence between groups. In the present context, some observers may be inclined to predict that xenophobia is positively (rather than negatively) associated with the intent to engage in behaviors that protect xenophobic individuals from infection by outgroup members. The present finding, therefore, underscores the maladaptive nature of the presently observed negative association. This is analogous to Rydgren’s (2004) assertion of xenophobic beliefs that “Although such beliefs are mostly non-rational from an objective perspective, because of their incongruence with reality, under certain conditions they may *appear* [our emphasis] rational from people’s *subjective* [our emphasis] point of view—in particular in situations of uncertainty.” To the extent that xenophobia relates to conspiratorial beliefs, it may also be linked to cognitive processes that appear to underpin conspiratorial thinking, with empirical evidence pointing toward disturbances in pattern recognition, agency attribution, coalition detection, and threat and secrecy detection (van Prooijen and van Vugt, 2018).

In contrast, the symptoms of negative affect were weakly but positively associated with pandemic health behaviors. This suggests that, at least in a non-clinical population, moderate (i.e., subclinical) degrees of negative affect may promote the pursuit of protective behaviors to mitigate the risk of infection. Interestingly, among the three negative affect syndromes, paranoia showed the strongest association with xenophobia and the strongest cross-loading onto the Maladaptive Belief factor. Paranoia is conceived of, to some extent, as a disturbance of both belief and affect, and by definition, it is directed at others in one’s environment (although not necessarily outgroup members). These features could explain why its correlation with xenophobia was intermediate between anxiety and depression on the one hand and conspiracy beliefs on the other. In addition, paranoia is generally conceived of as having a much more substantial negative impact on interpersonal function than anxiety or depression, and the present finding may form yet another expression of this important distinction among the three syndromes. It remains to be empirically determined whether xenophobia is heightened in clinical populations with functionally significant disturbances in affect (e.g., anxiety or mood disorders) or ideation (e.g., schizophrenia).

This study is cross-sectional in nature and lacks the explicit use of clinical diagnostic instruments. Nonetheless, the present findings suggest that among non-clinical populations, a fuller characterization of the basis for xenophobia, its expression, and its exacerbation during pandemics may warrant investigation of how these beliefs are formed, including the identification of a common underlying basis for xenophobia and conspiratorial beliefs. For instance, there remains a paucity of empirical research into precisely how the Behavioral Immune System may influence belief formation in response to the threat of contagion, in contrast to affective expression in response to this threat. In addition, interventions that aim to mitigate the varied deleterious effects of xenophobia may need to target the altered beliefs that support or exacerbate xenophobia, rather than solely targeting negative affect. These strategies may benefit from adapting those proposed to address and counter the psychological effects of disinformation (Peng et al., 2023). The likelihood of success in this endeavor remains uncertain. However, given the immense global consequences of xenophobia, further efforts are warranted (Abubakar et al., 2022; Peng et al., 2023).

## Data Availability

The raw data supporting the conclusions of this article will be made available by the authors, without undue reservation.
